# Targeted Printing of Cells: Evaluation of ADA-PEG Bioinks for Drop on Demand Approaches

**DOI:** 10.3390/gels8040206

**Published:** 2022-03-24

**Authors:** Emine Karakaya, Faina Bider, Andreas Frank, Jörg Teßmar, Lisa Schöbel, Leonard Forster, Stefan Schrüfer, Hans-Werner Schmidt, Dirk Wolfram Schubert, Andreas Blaeser, Aldo R. Boccaccini, Rainer Detsch

**Affiliations:** 1 Department of Materials Science and Engineering, Institute of Biomaterials, Friedrich-Alexander University Erlangen-Nürnberg, Cauerstraße 6, 91058 Erlangen, Germany; emine.karakaya@fau.de (E.K.); faina.bider@fau.de (F.B.); lisa.schoebel@fau.de (L.S.); aldo.boccaccini@fau.de (A.R.B.); 2 Macromolecular Chemistry I and Bavarian Polymer Institute (BPI), University of Bayreuth, Universitätsstrasse 30, 95447 Bayreuth, Germany; andreas.frank@uni-bayreuth.de (A.F.); hans-werner.schmidt@uni-bayreuth.de (H.-W.S.); 3 Department of Functional Materials in Medicine and Dentistry and Bavarian Polymer Institute (BPI), University of Würzburg, Pleicherwall 2, 97070 Würzburg, Germany; joerg.tessmar@fmz.uni-wuerzburg.de (J.T.); leonard.forster@fmz.uni-wuerzburg.de (L.F.); 4 Department of Materials Science and Engineering, Institute of Polymer Materials, University Erlangen-Nürnberg, Martenstraße 7, 91058 Erlangen, Germany; stefan.schruefer@fau.de (S.S.); dirk.schubert@fau.de (D.W.S.); 5 Bavarian Polymer Institute, Key Lab Advanced Fiber Technology, Dr.-Mack-Straße 77, 90762 Fürth, Germany; 6 Department of Mechanical Engineering, BioMedical Printing Technology, Technical University of Darmstadt, Magdalenenstr. 2, 64289 Darmstadt, Germany; blaeser@idd.tu-darmstadt.de; 7 Centre for Synthetic Biology, Technical University of Darmstadt, Schnittspahnstr. 10, 64287 Darmstadt, Germany

**Keywords:** bioprinting, drop on demand, sodium alginate, polyethylene glycol, shear stress

## Abstract

A novel approach, in the context of bioprinting, is the targeted printing of a defined number of cells at desired positions in predefined locations, which thereby opens up new perspectives for life science engineering. One major challenge in this application is to realize the targeted printing of cells onto a gel substrate with high cell survival rates in advanced bioinks. For this purpose, different alginate-dialdehyde—polyethylene glycol (ADA-PEG) inks with different PEG modifications and chain lengths (1–8 kDa) were characterized to evaluate their application as bioinks for drop on demand (DoD) printing. The biochemical properties of the inks, printing process, NIH/3T3 fibroblast cell distribution within a droplet and shear forces during printing were analyzed. Finally, different hydrogels were evaluated as a printing substrate. By analysing different PEG chain lengths with covalently crosslinked and non-crosslinked ADA-PEG inks, it was shown that the influence of Schiff’s bases on the viscosity of the corresponding materials is very low. Furthermore, it was shown that longer polymer chains resulted in less stable hydrogels, leading to fast degradation rates. Several bioinks highly exhibit biocompatibility, while the calculated nozzle shear stress increased from approx. 1.3 and 2.3 kPa. Moreover, we determined the number of cells for printed droplets depending on the initial cell concentration, which is crucially needed for targeted cell printing approaches.

## 1. Introduction

Three-dimensional (3D) bioprinting is a promising technology uniting the areas of biological, chemical and process engineering to create functional tissues mimicking the structure and characteristics of natural tissue for pharmaceutical and medical applications [1,2,3,4,5]. In recent years, various bioprinting techniques have been developed, allowing the design and fabrication of complex 3D structures by printing biological molecules, such as living cells, in numerous biomaterials [1,6,7]. In this regard, an important approach is the targeted printing of cells at desired positions on predefined locations, enabling new possibilities and perspectives in life science engineering [1,8,9]. For this purpose, cells are embedded in hydrogel precursor solutions forming a bioink, which is subsequently printed in a liquid like state on a substrate followed by a treatment using a crosslinker, leading to the gelation of the hydrogel and finally providing dimensionally stable 3D architectures on the printing substrate [10,11,12,13]. Compared to the common bottom-up technique, this top-down method can be used for the targeted printing of cells on various printing substrates, such as living tissues, 3D printed scaffolds or electrically conductive chips [14,15]. However, this technique is quite challenging and requires the use of a precise tailor-made 3D printer, a suitable hydrogel precursor for cell printing as well as a functional material providing a stable substrate. The drop on demand (DoD) method can be applied in this regard, representing a well-known 3D printing technique, which may fulfil the crucial task of controlled cell placement on biomaterials [10,16,17,18]. Other advantages brought by the DoD technology are the controlled ejection of small droplet volumes (150 pL–1 µL), improving the printing precision significantly and lowering the bioink consumption at the same time [19,20,21]. Moreover, the chosen material used as a bioink for targeted printing approaches needs to exhibit further suitable rheological properties, providing a homogeneous distribution of embedded cells during printing and the maintenance of 3D droplet structures during incubation [22,23,24]. Sodium alginate, a natural based hydrogel, is a favourable material for biofabrication because of its biocompatibility, mild gelation with divalent ions as well as chemical tenability. It, therefore, can be considered a suitable basis for the development of advanced bioink formulations [7,25]. Even more, the polysaccharide can be oxidized to alginate dialdehyde (ADA), which enables the formation of Schiff’s base with further amine-containing compounds, including proteins [26,27,28,29,30]. These newly introduced compounds may improve the properties of an alginate, yielding enhanced cell–material interactions. In most of the studies so far, proteins such as gelatin (GEL) were covalently bond to ADA via Schiff’s base formation, to improve the cell binding ability of the polysaccharide [25,26,30]. However, the natural source of these proteins, as well as their sensitive thermogelling behaviour, might be challenging for the reproducibility of their properties for specific applications [31]. Therefore, synthetic polymers such as modified polyethylene glycol (PEG), being able to mimic the biochemical properties of proteins, can be used in order to improve the ink properties of alginate-based materials [32,33]. PEG is a biocompatible synthetic polymer exhibiting terminal hydroxyl groups (PEG-diol) in its common form, which can be easily converted, further, to, for example, PEG-diamine containing two terminal amine groups for possible covalent binding to aldehyde groups of ADA under the formation of a Schiff’s base [34]. To the best of the author’s knowledge, the combination of ADA and PEG-diamines, namely, ADA-PEG, represents a novel bioink composition exhibiting further promising chemical flexibilities depending on further PEG modifications. Furthermore, the cell–material interactions, as well as the precise positioning of cells, can be, additionally, optimized not only by the choice of bioink but also by the application of soft materials as printing substrates [10]. Recent investigations showed that cells printed on wet and soft substrates exhibited a significantly improved cell viability by reducing possible cell stress [35]. Therefore, hydrogels can also be considered as promising candidates serving as printing substrates, due to their soft and porous nature which can be further modified by the polymer and crosslinker concentration [7,30]. Moreover, these soft materials can be locally immobilized by further crosslinking, which enables the accurate placement of cells.

The main goal of the present work was the realization of the targeted printing of cells on a gel substrate yielding high cell survival rates using NIH/3T3 fibroblast cells in ADA-PEG bioinks. For this purpose, different ADA-PEG inks with different PEG modifications and chain lengths (1–8 kDa) were characterized to evaluate their application as bioinks for DoD approaches. In this context, the biochemical properties of the inks and printing properties, such as cell distribution within a droplet, printing accuracy, shape fidelity and shear forces during printing, were analysed. Lastly, hydrogels, e.g., ADA-GEL, Pluronic F-127 and human platelet lysate (HPL), were used as printing substrates instead of pure polystyrene (PS). The hydrogels were investigated in terms of the cell survival of printed fibroblasts in order to evaluate the optimal material that can serve as an ideal substrate for targeted cell printing approaches.

## 2. Results and Discussion

### 2.1. Bioink Synthesis and Characterization

**Figure 1:**
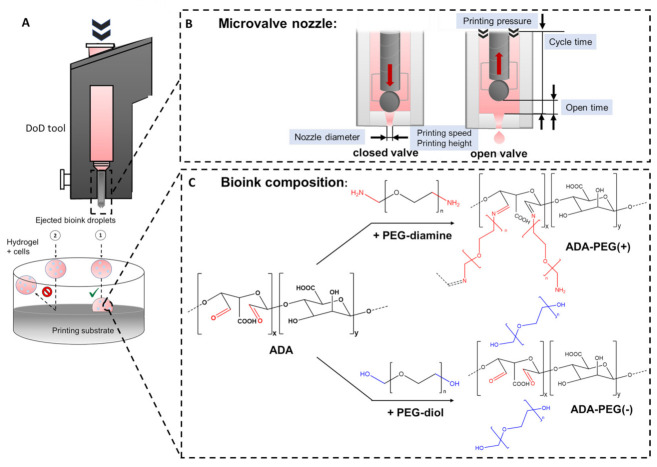
(**A**) Graphical illustration of a DoD tool and an attached (1, right) and unattached (2, left) ejected droplet on a printing substrate after ionically crosslinking with CaCl_2_; (**B**) variable printing parameters of the DoD printer influencing droplet formation: nozzle diameter, printing pressure, open time, cycle time, printing speed and printing height; (**C**) chemical structures of starting materials ADA and PEG-diamine leading to covalently crosslinked ADA-PEG(+) and chemical structures of ADA and PEG-diol yielding in covalently uncrosslinked ADA-PEG(-) products.

Bioinks used in DoD approaches must meet some requirements to work as suitable candidates for 3D printing purposes. Due to the small needle sizes used for DoD approaches, suitable bioinks are restricted to materials with low viscosities [10]. Moreover, the chosen bioink must enable a homogenous distribution of cells and, additionally, maintain the printed 3D shape after printing, which is realized by a possible gelation [8]. Since the aim of the present work was to print cells encapsulated in a hydrogel at defined locations on certain substrate surfaces, the printed droplets should also adhere to the surface to prevent the formation of floating hydrogel spheres (Figure 1A).

Therefore, a further crosslinking at the interface between droplet and substrate surface is desirable, enabling the additional fixing of the printed droplet on the surface. This approach was realized using alginate based materials as both bioink and printing substrate because of the chemical tunability of the polysaccharide determining the crosslinking efficiency with divalent cations. For instance, calcium ions (Ca^2+^) can react with glucuronic acid (G) blocks within the alginate chains, causing the formation of a stable polymer network, whereas the stiffness and stability of the hydrogel are influenced by the concentration of the crosslinker as well as the polymer [30]. By varying these material characteristics and printing parameters as open time, cycle time, pressure and speed (Figure 1B), it is possible to yield stable droplets, exhibiting a further crosslinking at the interface holding the cells in hydrogel droplets on the substrate in place. Therefore, it might be possible to use alginate based bioinks (Figure 1C) to improve printing precision by enhancing the shape fidelity on the printing substrate.

#### 2.1.1. Chemical Composition

A combination of oxidized alginate and linear PEGs (PEG-diol or PEG-diamine) with an approximate chain length of either 1, 4 or 8 kDa, namely, ADA-PEG(-) and ADA-PEG(+), was chosen to develop bioinks to investigate the effect of the PEG chain length as well as the influence of the presence of additional covalent crosslinking of ADA-PEG(+) inks on printing fidelity and cell survival (see Figure 1C). Firstly, a defined amount of hydroxyl groups of alginate monomers was oxidized yielding ADA with a degree of oxidation (%DO) of approximately 13%. Further, the hydroxyl groups of PEG-diols were modified to PEG-diamine using a two-step synthesis. The hydroxyl end groups of PEG-diols were activated with *p*-toluenesulfonyl chloride (TsCl) to yield the PEG-ditosylate intermediates and then converted with ammonia to amino end groups (see Figure S1). ^1^H-NMR measurements were conducted to confirm the successful transformation to amino end groups. The complete conversion is exemplarily shown for PEG-diamine 4 kDa by the disappearance of the aromatic signals of the PEG-ditosylate at 7.8 and 7.3 ppm (c and d), the triplet at 4.1 ppm (e) and the singlet at 2.4 ppm (f) and the appearance of the triplet at 3.0 ppm (g), as shown in Figure S2. Furthermore, the number average molecular weight (M_n_) of the synthesized PEG-diamines was determined using a standard potentiometric titration method. The investigations of the end group titration revealed an M_n_ of 1220 g·mol^−1^ for PEG-diamine 1 kDa, 3877 g·mol^−1^ for PEG-diamine 4 kDa and 10,540 g·mol^−1^ for PEG-diamine 8 kDa, which shows that the data provided by the supplier were in the measured range.

**Figure 2:**
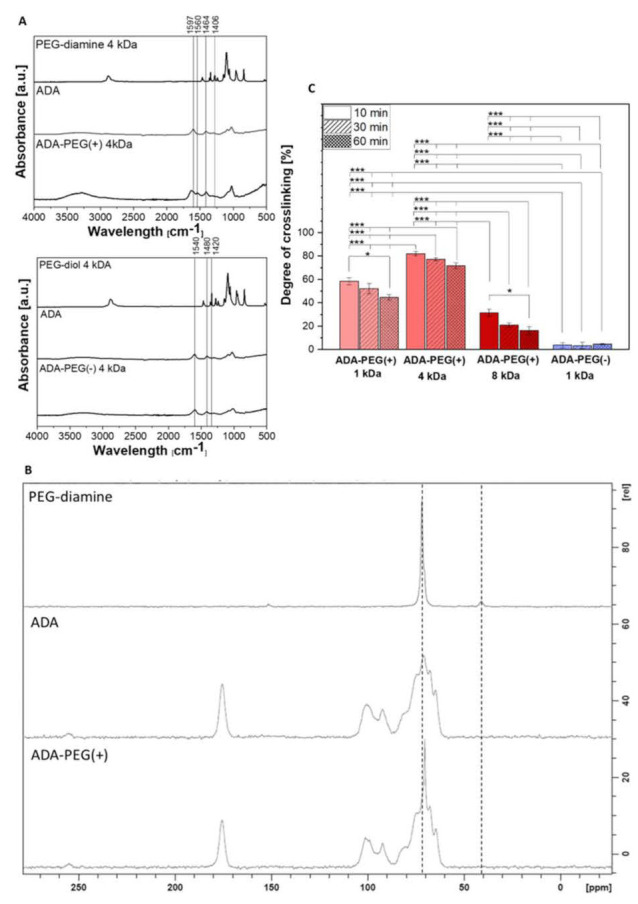
(**A**) FTIR spectra of ADA, PEG-Diol, PEG-Diamine and ADA-PEG inks with (+) or without (-) covalent bond; (**B**) ^13^C-solid state NMR spectra of ADA, PEG-diamine and ADA-PEG(+); (**C**) degree of crosslinking of ADA-PEG inks with (+) or without (-) covalent crosslinking after a reaction time of 10, 30 and 60 min (*n* = 5). ns * *p* < 0.05, ** *p* < 0.01, *** *p* < 0.001.

Finally, ADA was combined either with PEG-diamines (1–8 kDa) yielding an ink with a covalent crosslinking, namely, ADA-PEG(+) or with PEG-diols (1–8 kDa) leading to ADA-PEG(-) inks without a covalent crosslinking. The impact of the internal formation of Schiff’s base on the ink properties was in particular focus and, thus, investigated. In this regard, ATR-FTIR analysis was performed to investigate the covalent crosslinking between ADA and PEG-diamine, as reported earlier [26]. The absorption bands depicted in Figure 2A show characteristic stretching vibrations in the region of 1597–1540 cm^−1^ and 1406–1480 cm^−1^ for carboxylate groups (COO^−^) in ADA. No peak showing the symmetric vibration of aldehyde at approximately 1735 cm^−1^ could be obtained. The FTIR spectra of PEG-diol 4 kDa and PEG-diamine 4 kDa did not show any significant difference, which could be attributed to any chemical conversation of PEG. However, FTIR spectra of final compounds ADA-PEG(-) 4 kDa and ADA-PEG(+) 4 kDa differ essentially from each other. While the COO^−^ signal of ADA at 1540 cm^−1^ did not change after the addition of PEG-diol, the COO^−^ signal of ADA at 1597 cm^−1^ was shifted to 1615 cm^−1^ after the combination with PEG-diamine. Additionally, a further signal at 1560 cm^−1^ appeared in the ADA-PEG(+) spectrum, which could be related to C=N vibrations indicating covalent crosslinking between ADA and PEG-diamine. The absence of any C=N vibration peak around 1560 cm^−1^ in ADA-PEG(-) demonstrated that only a physical attraction prevails between ADA and PEG-Diol compounds and no covalent bond was formed.

Figure 2B depicts the solid state ^13^C NMR spectra of ADA, PEG-diamine 4 kDa and ADA-PEG(+) 4 kDa hydrogels after lyophilisation. The spectrum of ADA has already been extensively discussed and is well known from literature [26]. Furthermore, a signal at 40 ppm was observed in the PEG-diamine spectrum representing terminal amino groups (C-N). In this regard, the intensity of the signal at 73 ppm and 40 ppm may show a correlation between the amount of C-O bonds in the polymer chain and to the C-N bonds at the end groups. Moreover, the absence of the C-N signal at 40 ppm indicates a conversion of these functional groups, yielding imine groups. Nevertheless, no signal for a C=N carbon was found at 150 ppm in the ADA-PEG(+) spectrum. This suggests that amino groups do react with the aldehyde groups of ADA but the amount of formed imine bonds is relatively small, which is not detectable anymore. The fact that the signal disappears completely at 40 ppm also suggests that most of the amino groups contribute to the formation of Schiff’s base, which is in accordance with the FTIR results. The main part of the polymer chains consisting of homogenous C-O bonds remains unchanged after the chemical crosslinking with ADA, which is confirmed by the signal at 70–73 ppm in both spectra.

Moreover, results of the performed TNBS assay shown in Figure 2C revealed high values for the degree of crosslinking of all ADA-PEG(+) compounds after 10 min of stirring. In this regard, it is particularly noticeable that all values drop slightly after 30 min and show minimum values after a stirring time of 60 min. Furthermore, no significant crosslinking could be obtained for ADA-PEG(-) inks due to the lack of amine groups. Thus, it can be assumed that the crosslinking between amino and aldehyde groups of ADA-PEG(+) hydrogels takes place relatively quickly and is completed after 10 min. As a result, the maximal crosslinking could be achieved within 10 min, which has not been reported in literature so far. Since the formation of Schiff’s base is reversible and can regress, the results from Figure 2C suggest the breakage of the formed imine bond after 10 min, resulting in slightly lower crosslinking values for all compounds [36]. In addition, it is notable that ADA-PEG(+) 4 kDa has the highest degree of crosslinking for all time points. This may be explained by the electrostatic interaction between the polymer chains.

In the formation of a new covalent bond the reactivity of the functional groups, but also the electrostatic attraction or repulsion, plays an important role [37,38]. While ADA chains may repel each other due to similar charges, PEG-diamine compounds exhibiting amino groups attack the carboxyl carbons of ADA enabling the formation of Schiff’s bases. In addition, polymer chains of PEG contain repeating units of ethylene oxides groups which can slightly form hydrogen bonding with COO^−^ groups of ADA. The results suggest that the PEG-diamines 4 kDa represents an ideal remedy in terms of polymer interactions between ADA and PEG, resulting in statistically higher crosslinking values for ADA-PEG(+) 4 kDa inks in contrast to the rest. It seems like a PEG chain length of 4 kDa is sufficient to fit between ADA chains enabling the crosslinking of more terminal amine groups yielding a maximum crosslinking.

#### 2.1.2. Rheological Characterization

**Figure 3:**
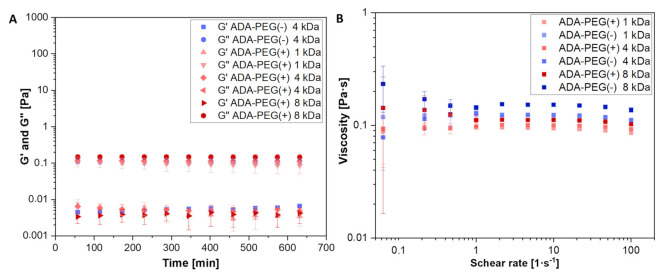
(**A**) Oscillatory time-sweep study for the measurement of G″ (above) and G′ (below) of ADA-PEG(+) and ADA-PEG(-) inks under constant shear rates at 37 °C; (**B**) continuous flow sweep for measuring viscosity of ADA-PEG(+) and ADA-PEG(-) inks for a flow sweep up to 100 s^−1^ (*n* = 3).

For the preparation of good shape fidelity bioinks, rheological properties need to be fully investigated in detail to assess the impact of material characteristics and chemical properties on printability as well as cell survival [39]. Since it is known that the formation of Schiff’s base may affect the rheological properties of the hydrogel [40], the influence of additional covalent bonds between ADA and PEG-moieties on rheological behaviour were investigated by comparing the covalent crosslinked ADA-PEG(+) hydrogels with the uncrosslinked ADA-PEG(-) equivalents. Figure 3A depicts the storage (G′) as well as the loss modulus (G″) of these hydrogel precursors, enabling an investigation of their rheological behaviour. Noteworthy is that no difference between the crosslinked (+) and uncrosslinked ADA-PEG(-) hydrogel precursors was obtained, as the ratio of G′ to G″ of the samples did not change over a period of 10 min. Furthermore, G″ dominated the rheological behaviour of all ADA-PEG samples in the tested regime, underlining the rheological liquid like material behaviour. Thus, the crosslinking between ADA and PEG-diamine could not be confirmed since no increase in the corresponding ADA-PEG(+) modulus was observed compared to ADA-PEG(-) inks. Furthermore, the zero shear viscosities of all hydrogels were investigated at varying flow sweeps between 0.1 and 100 s^−1^, as depicted in Figure 3B. First, the tested bioinks did not show a strong dependency of the viscosity on the shear rate in the tested regime. In this regard, it is notable that all viscosities did not differ greatly from each other and they were in the range from 0.09 to 0.16 Pa·s. This behaviour could be the reason why no indication for the formation of a covalent crosslinking for ADA-PEG(+) inks could be observed, since minor differences in this region may not be detectable by the used rheometer. However, the rheological nature of these hydrogels makes them ideal candidates for DoD printing approaches, which require rheological liquid like and low viscous materials [8]. Therefore, all covalently crosslinked (+) and uncrosslinked (-) ADA-PEG hydrogels are highly suitable for application in DoD approaches.

#### 2.1.3. In Vitro Swelling and Degradation Behaviour

**Figure 4:**
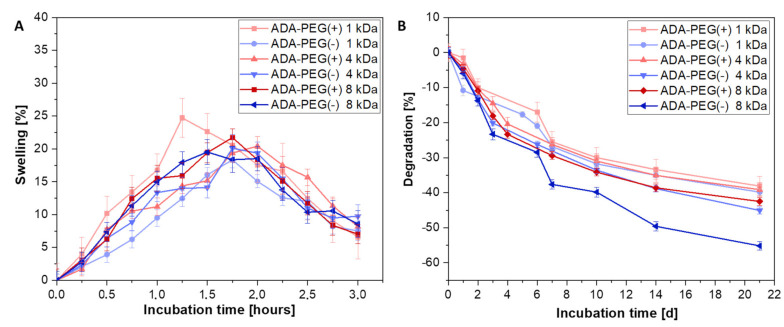
(**A**) Swelling and (**B**) degradation behaviour of ADA-PEG inks incubated in HBSS at 37 °C for 21 days (*n* = 6).

The ink properties after cell printing are highly important as well as the material properties investigated prior bioprinting. Following the targeted placement of cells on the printing substrates, the bioink should maintain its function and provide certain protection to the cells during incubation and medium changes. In this regard, the rate of degradation of the bioink plays an important role and should be studied in detail, enabling an application specific decision depending on the incubation time. Therefore, inks with a long term stability for at least 3 weeks were required. Since alginate based hydrogels can be easily modified—for example, by oxidation to ADA—it is possible to vary the composition in such way that the desired degradation rate was reached. For this reason, all ADA-PEG(+) and ADA-PEG(-) hydrogels with PEGs of different chain lengths (1–8 kDa) were investigated after crosslinking with CaCl_2_ in terms of swelling and degradation (see Figure 4) to analyse the material behaviour during the incubation in the cell culture medium. Within the first 2 h of incubation, a weight gain resulting in swelling of approximately 20% is clearly shown for all compositions. Subsequently, a weight loss is depicted which is normalized to the initial value within the first 24 h. In this period, the observed water that was stored between the ADA and PEG chains was released to the medium yielding in the initial weight of the hydrogel films. No significant trend among the different ADA-PEG inks and the respective PEG compounds was observed. This is because the chemophysical properties of ADA are superior due to their high polymer amount compared to PEG compounds in ADA-PEG(+) and ADA-PEG(-). Therefore, it can be concluded that the swelling behaviour of ADA dominated and, for this reason, no influence of Schiff’s base or PEG chain length could be observed on the swelling behaviour within the first 2 h for all compositions. After 24 h, the degradation of all materials started and a certain trend depending on PEG choice was obtained (see Figure 4B). It is particularly noticeable that the graph of ADA-PEG(-) (1–8 kDa) indicates that the degradation also increases with increasing PEG chain length. In this regard, there is no chemical crosslinking between ADA and the PEG-diol moieties and, therefore, the only crosslinking enabling a dense network is due to Ca^2+^ ions yielding a physical crosslinking of the ADA chains. Based on the results of Figure 4B shorter PEG-diols (1 and 4 kDa) seem to be included more efficiently in the final network resulting in a slower degradation over 21 days. However, even though a covalent crosslinking is present in ADA-PEG(+) inks, this does not seem to have a major influence on the degradation over 21 days, as they show a similar linear trend over the whole incubation time compared to ADA-PEG(-) inks. However, it is important to highlight that ADA-PEG(-) 8 kDa show significantly higher degradation resulting in a weight loss of 60% compared to the covalently crosslinked equivalent, ADA-PEG(+) 8 kDa, where a weight loss of 40% was observed illustrating the strong influence of Schiff’s base on the long term stability of the final construct. Nevertheless, it is also clearly observable that the influence of the physical crosslinking with Ca^2+^ compared to the chemical crosslinking is dominant, determining mainly the stability of the inks.

#### 2.1.4. Mechanical Analysis Using Compression Tests

**Figure 5:**
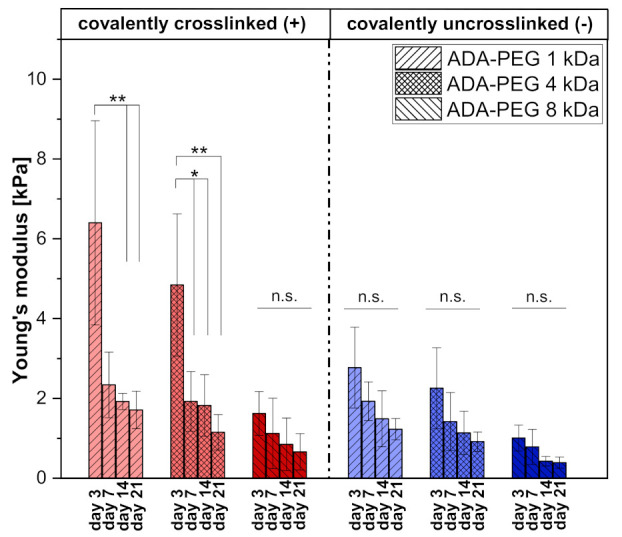
Mechanical analysis of ADA-PEG inks after crosslinking with CaCl_2_. Compression stress studies revealing Young’s moduli of crosslinked (+) and uncrosslinked (-) ADA-PEG inks over an incubation time of 21 days incubated at 37 °C in a controlled atmosphere of 5% CO_2_ and 95% relative humidity. All data is displayed as SD. ns * *p* < 0.05, ** *p* < 0.01, *** *p* < 0.001.

Figure 5 depicts the average Young’s moduli of covalently crosslinked (+) and uncrosslinked ADA-PEG(-) inks for 3, 7, 14 and 21 days of incubation at ambient temperatures. The results indicate a correlation between Young’s moduli and PEG chain length. As already shown in the degradation studies, the degradation and, thus, the stability of the constructs of ADA-PEG(+) and ADA-PEG(-) hydrogels decreased with increasing PEG chain length. In this context, it can be clearly seen that polymer networks containing PEGs with a chain length of 1 kDa showed the highest Young’s moduli, whereas ADA-PEG inks containing PEGs with a chain length of 8 kDa exhibited the lowest stiffness (see Figure 5). A possible explanation for this trend is the porosity of the hydrogels. It is known that the porosity of PEG containing hydrogels increases with longer PEG chain lengths attributed to the strength of the crosslinking [41]. The longer the PEG chains are, the bigger is the space between the ADA strands crosslinked with divalent ions, which leads to a network with spatial gaps. These cavities between the polymer strands lead to material porosity, influencing the Young’s moduli. Due to higher pore contents and pore sizes and, thus, lower crosslinking strengths, the entire network system becomes more unstable and softer, resulting in a decrease in stiffness [42]. Furthermore, comparing the Young’s moduli of covalently crosslinked (+) with the uncrosslinked (-) ADA-PEG systems, a similar range for all values is found, hardly differing from each other over an incubation of 21 days. This confirms that the chemical crosslinking does not influence the mechanical properties of the hydrogels significantly, revealing the superior influence of the physical crosslinking with Ca^2+^ ions on the stability as well as stiffness of the material. This observation can be explained by the interaction of Ca^2+^ ions with G-blocks, which are still predominantly present in ADA. Previous studies proved that, during the oxidation of alginate, only G-blocks are attacked by NaIO_4_ due to their steric position [43]. However, depending on the amount of oxidation reagent used, still a high number of G-blocks remains in each alginate chain, which can be attacked by Ca^2+^ ions to form a stable polymer network. Since the present ADA exhibits only 13% of aldehyde groups, the effect of the formed Schiff’s bases with this amount of generated functional groups is negligible, in comparison to the physical crosslinking induced by Ca^2+^ ions and, as a result, it had no influence on the Young’s moduli of the hydrogels.

### 2.2. Evaluation of the Printing Process

#### 2.2.1. Analytical Modulation of the Printing Process

In order to evaluate the influence of the hydrogel’s rheological properties on the printing process, the fluid flow within the microvalve during drop based dispensing was analytically analysed according to a previous reported methodology [44]. In detail, the results provide insight into the estimated droplet ejection speed and the average nozzle shear stress (see Table 1). The analysis was conducted for the three hydrogel formulations investigated in this study (ADA-PEG(+) 1 kDa, ADA-PEG(+) 4 kDa, and ADA-PEG(+) 8 kDa). As expected, the consistency factor (*k*) of the gels increased with increasing molecular weight of the applied PEG-diamine, from 20.9 mPa·s (ADA-PEG(+) 1 kDa) to 43.4 mPa·s (ADA-PEG(+) 8 kDa). The flow exponent (*n*) of the analysed gels indicated a slight shear thinning behaviour ranging from 0.96 to 0.98.

In accordance with the distinct consistency factors, with the increasing molecular weight of the added PEG the estimated droplet ejection speed decreased from 7.35 m·s^−1^ to 5.94 m·s^−1^, while the average nozzle shear stress increased from 1.3 to 2.3 kPa.

As expected due to the different rheological properties, the calculated shear stress values differ significantly from each other. However, even with ADA-PEG(+) 8 kDa they are clearly below the threshold of 5 kPa, which is considered critical for printing living cells [44]. For this reason, no impairment of cell viability or cell functionality is to be expected from the shear stress alone, in all three cases.

The calculated difference in droplet exit velocities is also expected in this context. This, too, can possibly affect the vitality and functionality of printed cells, since the cells enclosed in the droplets are subjected to different impulses upon impact with the substrate. There is, as yet, no comprehensive literature data on this. However, the data collected in this study indicate that the stiffness of the substrate plays a significant role in the transmission of the impulse to the cells. Cells printed on PS substrates enclosed in low molecular weight, ADA-PEG(+) 1 kDa, for example, exhibit significantly lower viability than cells integrated in higher molecular weight ADA-PEG (analysis in Figure 7C). The observed effect can be attributed to the different exit velocities. Further studies should be performed to clarify this issue in the future.

#### 2.2.2. Evaluation of Printing Parameters

Since the main objective of this research is the deposition of cells at desired positions using the DoD technology, it is necessary to understand the function of the printer by evaluating the impact of the set parameters on the geometry of the printed scaffold. This should be performed for every bioink individually, enabling the use of this technology in an application specific manner. To evaluate the use of ADA-PEG hydrogels as bioinks, only covalently crosslinked ADA-PEG(+) inks with different PEG chain length (1–8 kDa) were chosen.

**Figure 6:**
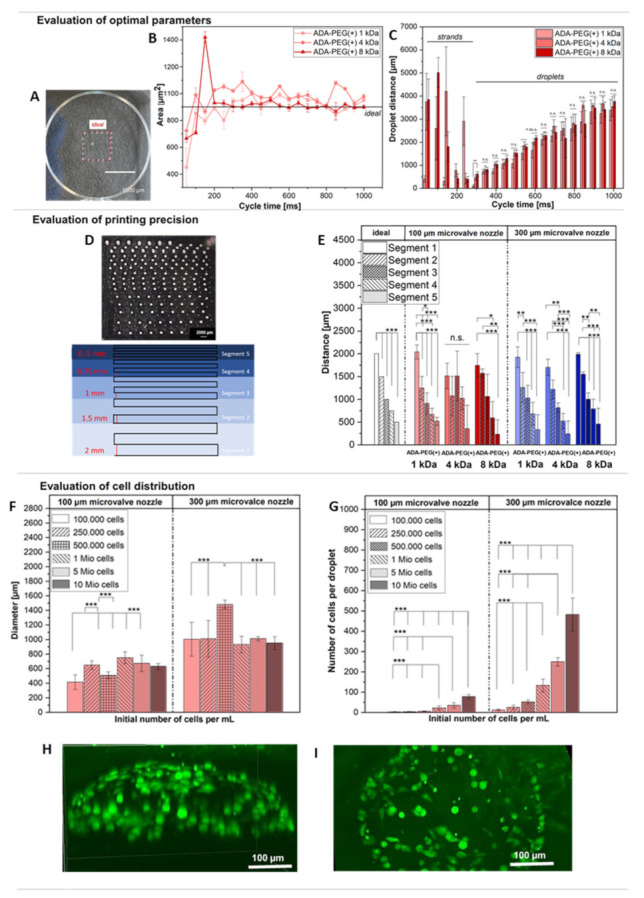
(**A**) Image of printed ADA-PEG(+) 1 kDa droplets using a square rectangle template with dimensions of 10 × 10 mm on PS with an added red square representing borders of the simulated ideal area. Scaffolds were printed using a 300 µm microvalve nozzle (*n* = 6); (**B**) influence of cycle time on the printing precision of a printed square shaped scaffold using ADA-PEG(+) 1, 4 and 8 kDa inks determined by the overlapping of the stimulated area with the printed one. Scaffolds were printed using a 300 µm microvalve nozzle (*n* = 6); (**C**) influence of the cycle time on droplet distances using ADA-PEG(+) 1, 4 and 8 kDa inks printed on PS. Scaffolds were printed using a 300 µm microvalve nozzle (*n* = 6); (**D**) illustration of the resolution tree for the filament fusion test showing segments 1–5 (bottom) and a light microscopy image of the printed version using ADA-PEG(+) 1 kDa on PS (up); (**E**) diagram showing the distances between the segments 1–5 of the printed resolution tree for filament fusion tests of ADA-PEG(+) 1 kD, 4 kDa and 8 kDa on PS. Scaffolds were printed using 100 and 300 µm microvalve nozzles (*n* = 2); (**F**) determined diameters of printed ADA-PEG(+) 1 kDa droplets containing NIH/3T3 cells (100,000–10 Mio·mL^−1^) using 100 µm and 300 µm microvalve nozzles (*n* = 20); (**G**) determined cell concentrations per printed ADA-PEG(+) 1 kDa droplet containing NIH/3T3 cells (100,000–10 Mio·mL^−1^) using 100 µm and 300 µm microvalve nozzles (*n* = 20); (**H**) confocal image of Calcein-stained NIH/3T3 cells (1 Mio·mL^−1^) in printed ADA-PEG(+) 1 kDa droplets on HPL: view z-direction and (**I**) view from top. ns * *p* < 0.05, ** *p* < 0.01, *** *p* < 0.001.

In a first approach, the working principle of the DoD printer was investigated using the 300 µm microvalve nozzle, and optimal parameters for a highly precise droplet printing were evaluated for every composition. In this regard, ADA-PEG(+) inks were used for printing a defined template showing a square rectangle geometry, depicted in Figure 6A, on PS, varying only the cycle time and the pressure. Table S2 shows a certain range for the applied printing pressures (15–40 kPa), which does not reveal any trend in correlation to the PEG chain lengths. This behaviour is in accordance with the obtained rheological results, which also did not show any significant trend (Figure 5). Based on the deviation of the printed area from the STL template (ideal: 100 µm^2^) an attempt was made to determine the printing precision. Thus, the accuracy of the DoD process was defined by the overlapping of the simulated area with the printed area, in which the area content of the printed scaffold was calculated. Each drop that has not been printed along the strand shows up as a deviation in Figure 6B. Even if no particular trend between the compositions could be observed, it can be observed that ADA-PEG(+) 8 kDa led to the highest printing precision with the least deviation for nearly all cycle times. Moreover, it should be mentioned that the number of droplets per strand could not be defined within the G-code and it was, rather, empirically determined by varying the cycle time. Figure 6C depicts no significant differences between the three ink compositions for the droplet distances. Further, it is notable that the standard deviation for cycle times below 300 ms is comparably high, which can be attributed to the merging of printed droplets. The printed drops had a very small distance between each other and, therefore, they unite after printing, resulting in the formation of large droplets with random diameters. From 300 ms onwards, a clear correlation between opening time and drop distances can be seen. Thus, with increasing opening time, the drop distance increases significantly. However, it should be mentioned that cycle times higher than 700 ms led to satellite droplets and negatively affect the printing precision. Therefore, cycle times from 700 ms onwards are rather unfavourable for DoD printing. Thus, it can be concluded that open times between 300–700 ms were suited for DoD printing of ADA-PEG(+) bioinks without any significant impact on the PEG chain lengths. Nevertheless, a cycle time of 400 ms was chosen to be ideal for the printing of a square rectangle with an area of 100 µm^2^ with the smallest possible droplet distances without merging. Therefore, for all following experiments, a cycle time of 400 ms was chosen to be ideal for all ADA-PEG(+) inks.

A further approach revealing an insight into the printing accuracy of the DoD printer is the filament fusion test, which was applied for all inks using a 100 and a 300 µm microvalve nozzle to evaluate the effect of nozzle diameter on ADA-PEG(+) inks. Figure 6D shows an illustration of five different segments with strut distances ranging between 500–2000 µm and an ejected corresponding resolution tree, according to Cai et al. [45], composed of ADA-PEG droplets on PS. The results of these printing accuracy tests are summarized in Figure 6E, where significantly higher standard deviations in all printed segments were obtained for the 100 µm microvalve nozzle compared to printed segments using the 300 µm one. One reason for the high deviations of the 100 µm nozzle compared to the 300 µm nozzle could be small blockages, which can easily deflect the droplet jet during printing, which only becomes visible by a shifted positioning of the droplets after printing on PS. With the 300 µm microvalve nozzle, this is less likely due to the larger nozzle diameter. Further, it can be observed that the determined distances of all segments of the 100 µm microvalve nozzle varied widely from the ideal simulated ones. In contrast to that, the distances of the segments 1–3 of the resolution trees printed with the 300 µm nozzle were in the expected regions and did not vary highly from the ideal values. Only for the segments 4 and 5, were high standard deviations as well as discrepancies in distances obtained. The main reason for this behaviour were merging droplets that occurred due to the significantly small distances (500–750 µm) between the segment struts. This experiment clearly demonstrated that droplet distances of up to 2000 µm could be successfully achieved using both nozzles for the distances in the 750–1500 µm range. In addition to that, it was clearly shown that the 300 µm nozzle showed a higher printing precision and was, therefore, recommended for targeted cell printing approaches.

In a last experiment, the number of cells per droplet was determined for the 100 and 300 µm microvalve nozzle to ensure a controlled placement of predefined cell numbers. Therefore, ADA-PEG(+) 1 kDa bioinks with different initial cell concentrations (100,000–10 Mio·mL^−1^) were used for ejecting droplets on PS (*n* = 20), whereas all other printing parameters were kept constant. Firstly, the diameters of the droplets were analysed to investigate the printing reproducibility of printed hydrogel spheres as well as to evaluate the cell distribution within them. Figure S3 depicts light microscopy images of individual droplets printed with a microvalve needle exhibiting a diameter of 100 µm (upper row) and 300 µm (lower row) and a cell density ranging from 100,000 to 10 Mio·mL^−1^. The images illustrate round shapes for all bioinks with homogenous cell distributions. Further, Figure 6F depicts the determined droplet diameters of the ejected droplets with different cell concentrations. The results clearly showed that no significant correlation between initial cell density and droplet shape could be obtained, neither for the 100 nor for the 300 µm microvalve nozzle. Furthermore, it can be clearly observed that the printing reproducibility of the 300 µm microvalve nozzle was essentially higher since only the diameters of droplets containing 500,000 cells per droplet were remarkably larger and outstanding, with 1450 µm compared to the remaining cell concentrations, which showed droplet diameters of approximate 1000 µm. Lastly, all the obtained diameters of all cell concentrations were combined to determine the average diameter of an ejected droplet using the 100 or the 300 µm microvalve nozzle, respectively. A significant increase in the droplet diameter, from approximately 600 µm to 1100 µm, with increasing needle diameter, from 100 µm to 300 µm on average, of all analysed cell concentrations was observed (see Figure S4). Furthermore, the number of cells within an ejected droplet of a certain initial cell concentration was determined for the 100 and 300 µm microvalve nozzle and illustrated in Figure 6G. It could be observed that, with increasing diameters, the cell concentration per droplet also increased. Thus, for the initial highest cell concentration (10 Mio·mL^−1^) approximately 77 cells per droplet for the 100 µm nozzle and approximately 480 cells per droplet for the 300 µm needle could be observed. However, it should be noted that, with increasing number of cells, the standard derivation also increases. The reason for this is the uncontrollable high number of cells and the lack of homogeneous distribution of cells within the droplet. Therefore, bioinks with higher cell densities should be mixed more frequently to avoid the sedimentation of cells in the cartridge [46]. Furthermore, with an initial concentration of 500,000 cells·mL^−1^ for the 100 µm nozzle and 100,000 cells·mL^−1^ for the 300 µm nozzle, it was possible to print the smallest number of cells per droplet (1–5 cells per droplet), which may be useful for printing approaches with the aim of the targeted printing of single cells on substrates. Moreover, 3 dimesional (3D) confocal microscopy images of Calcein stained cells encapsulated in an ADA-PEG(+) 1 kDa bioink droplet after 24 h revealed a homogenous cell distribution for bioinks, with an initial cell concentration of 1 Mio·mL^−1^ (Figure 6H,I). Besides that, a significant high number of living cells could be obtained. Further, it can be stated that alginate based hydrogels were able to maintain their 3D structure for 24 h, which confirms the successful use of ADA-PEG(+) hydrogels as bioinks suitable for DoD, since they fulfil all suitable requirements, such as homogenous cell distribution, protection of cells, shape maintenance and high cell viability rates. However, the 300 µm microvalve nozzle showed an easier handling due to less clogging of the needle and was, therefore, chosen for the following cell experiments. In addition, as a result, the shear stress calculations analysed for the 300 µm microvalve nozzle can be correlated with the cell behaviour after printing.

#### 2.2.3. Targeted Cell Printing on Substrates

**Figure 7:**
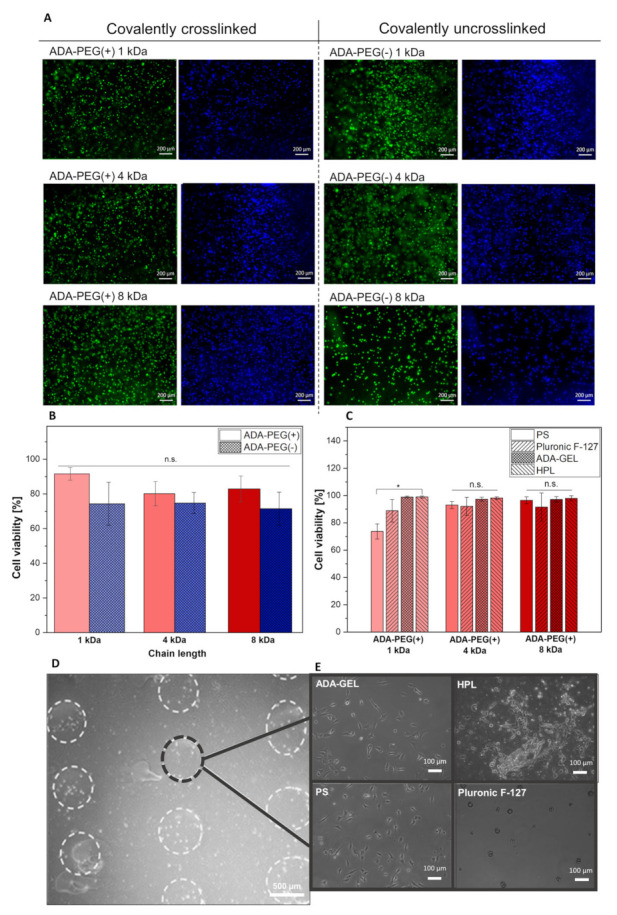
(**A**) Fluorescence images of NIH/3T3 cells embedded in covalently crosslinked (+) and uncrosslinked (-) ADA-PEG (1–8 kDa) bioinks stained with Calcein AM (green) and DAPI (blue); (**B**) cell viability of NIH/3T3 cells in covalently crosslinked (+) and uncrosslinked (-) ADA-PEG (1–8 kDa) bioinks after 24 h of incubation (*n* = 3); (**C**) cell viability of NIH/3T3 cells in covalently crosslinked ADA-PEG(+) bioinks printed on different printing substrates (PS, Pluronic F-127, ADA-GEL, HPL) after 24 h of incubation (*n* = 3); (**D**) image of ADA-PEG(+) 1 kDa droplets containing NIH/3T3 cells printed in parallel lines on a printing substrate using a 300 µm microvalve nozzle; (**E**) light microscopy images depicting fully stretched NIH/3T3 cells in ADA-PEG(+) 1 kDa droplets printed on ADA-GEL and HPL as well as NIH/3T3 cells in ADA-PEG(+) 1 kDa with round morphologies on PS and Pluronic F-127 used as printing substrates. ns * *p* < 0.05, ** *p* < 0.01, *** *p* < 0.001.

The use of different hydrogels as printing substrates were evaluated. Since several studies have shown that cells printed on a soft surface led to a higher cell viability rate for printed cells [47], soft biomaterials such as ADA-GEL, Pluronic F-127 and HPL were chosen as printing substrates. Figure 7A shows fluorescence images of NIH/3T3 cells stained with Calcein AM/DAPI embedded in ADA, covalently crosslinked (+) or uncrosslinked ADA-PEG(-) inks with different PEG chain lengths (1, 4 and 8 kDa) cultured for 24 h. It can be observed that with the addition of PEG-diamine to ADA, the viability of NIH/3T3 cells increased significantly from approximately 70% to >90%, in comparison to ADA-PEG(-) (see Figure 7B). What is notable in this regard is the increase in the cell viability of the fibroblasts in ADA-PEG(-) inks compared to the ones in ADA-PEG(+) inks, which can be directly linked to the influence of the formation of Schiff’s base as free and unbound aldehyde groups are known to be cytotoxic [26]. Due to the shielding of aldehyde groups caused by electrostatic interactions between ADA and PEG-diol compounds, the toxic aldehyde groups may have no direct contact to the fibroblasts, resulting in higher survival rates. With the introduction of covalently bound PEG-diamine moieties to the ADA system, the aldehyde groups are transformed into imine groups and, therefore, the highest cell viabilities were observed for ADA-PEG(+) hydrogels in comparison to pure ADA. For this reason, only ADA-PEG(+) hydrogels were used for further cell printing experiments where cell loaded bioinks were printed on PS, Pluronic F-127, ADA-GEL and HPL, to evaluate the most suitable printing substrate for DoD approaches, as shown in Figure 7C. This study shows a high cell survival rate for all compositions (>70%) printed on PS. Furthermore, a significant increase in the number of living cells in ADA-PEG(+) 1 kDa droplets could be observed when PS with an approximate cell compatibility rate of 70% was exchanged with a softer material, namely, Pluronic F-127, ADA-GEL, or HPL, all with cell survival rates over 88%. This trend was only slightly seen for NIH/3T3 cells in ADA-PEG 4 kDa and 8 kDa. Even though the results do not indicate a particular relation between PEG chain lengths and the survival rate, nevertheless, ADA-GEL and HPL showed the highest cell compatibility rates for all bioinks (approximate 98%). This confirms that softer substrates (Young’s modulus: 0–22 kPa [1]) in contrast to PS (Young’s modulus: 3250 MPa) lead to higher cell survival rates, which was also observed by Tirella et al. [35]. Comparing the cell viabilities of Figure 7B,C, showing both high viabilities for all investigated bioinks, it can be concluded that average nozzle shear stress within the 300 µm microvalve nozzle ranging from approx. 1.3 and 2.3 kPa calculated previously had no significant effect on the cell viability of fibroblasts after 24 h. This indicates the high biocompatibility of the DoD printing process using the 300 µm nozzle.

Figure 7D depicts a light microscopy image of the printed scaffold consisting of ADA-PEG(+) 1 kDa on ADA-GEL used as a printing substrate. It can be clearly seen that all droplets were in a line and even the round shapes of the droplets were still observable, which confirms a stable fixation of the droplets by ADA-GEL after incubation. This behaviour was also observable when HPL was used as a printing substrate. However, in the case of Pluronic F-127, the underlayer was partially dissolved during incubation and droplet spheres were floating, which contributes to bad fixation of the printed droplets. Lastly, the 2 dimensional (2D) morphology of NIH/3T3 cells in bioink droplets printed on the surfaces was investigated and depicted in Figure 7E. Significant spreading of fibroblasts was observed in light microscopic images of ADA-PEG(+) 1 kDa droplets printed on ADA-GEL and HPL both used as soft printing substrates. These improved cell–material interactions at the interface between droplets, and the printing substrates could not be observed for encapsulated NIH/3T3 cells in ADA-PEG(+) droplets on PS or Pluronic F-127 due to the lack of adhesive linkers in these printing substrates. Therefore, it can be concluded that all the investigated hydrogels are suitable printing substrates, showing highly promising results in terms of sufficient fixation of the droplets on their surface, leading to a high final printing accuracy. However, ADA-GEL and HPL are inducing cell-material interactions between the cells encapsulated in the hydrogel droplet on the corresponding substrate, yielding high cell survival rates.

## 3. Conclusions

In this study, we presented the targeted printing of NIH/3T3 fibroblast cells in alginate based hydrogels on various substrates using the DoD printing technology. The importance of this work lies in the evaluation of crucial parameters required for the success of the targeted placing of cells on predefined locations. For this purpose, suitable biomaterials serving as bioinks or printing substrates were investigated after obtaining an insight into the working principle of the scaffold generation using the DoD technology. In this regard, firstly, the interaction of different PEG chain lengths (1–8 kDa) with covalently crosslinked (+) and uncrosslinked ADA-PEG(-) inks were compared, to investigate the influence of the material composition on bioink characteristics. From a rheological point of view, no difference between all crosslinked (+) and uncrosslinked ADA-PEG(-) hydrogels were obtained, revealing the weak influence of Schiff’s base on the viscosity of the corresponding materials. These results confirmed, for the first time, the superior impact of physical crosslinking in contrast to the weak influence of the chemical crosslinking of partially oxidized alginate hydrogels on the measured viscosity. However, the effect of the covalent bond between ADA and PEG-diamine moieties was slightly observable in the long term stability studies of the hydrogels, which is an important criterion playing a key role in the protection of cells present on the surface of substrates cultivated for long periods of time. In comparison to the covalently uncrosslinked (-) inks, the crosslinked (+) ones showed a slightly slower degradation behaviour over 21 days. Further, the results showed that longer polymer chains led to less stable hydrogels, resulting in fast degradation rates. Mahendra et al. [41] assessed the correlation between long PEG chains and high porosity. We could confirm those results for ADA-PEG inks consisting of 8 kDa PEGs, where we observed higher degradation rates as well as low stiffnesses compared to the inks with 1 kDa or 4 kDa PEGs. Both material properties can be linked to the presence of particularly large pores [41] in the networks containing 8 kDa PEGs independently from the covalent crosslinking in ADA-PEG(+) and ADA-PEG(-) inks. Based on the promising results, only chemically crosslinked ADA-PEG(+) bioinks were used for the evaluation of printing parameters. We were able to show that these bioinks were highly biocompatible, while the average nozzle shear stress increased from approx. 1.3 and 2.3 kPa. In addition to that, these hydrogels were promising candidates for DoD approaches, fulfilling all the requirements needed for this technology. Using ADA-PEG(+) hydrogels, we were able to evaluate printing parameters and obtained high printing precision for all investigated bioinks independently from the PEG chain lengths. Moreover, we determined the number of cells for printed droplets depending on the initial cell concentration (100,000–10 Mio·mL^−1^), which is crucially needed for the prediction of the cell concentration in printed droplets for targeted cell printing approaches. However, detailed aspects of the bioink droplet, such as pore size, pore distribution and compressibility, will be part of future work. Finally, we evaluated ideal printing substrates, namely, ADA-GEL and HPL, which were able to increase the cell compatibility of printed NIH/3T3 cells using the DoD printing technology. Additionally, promising cell–material interactions on the surface of both hydrogels were found and spreading cells were observed. Therefore, ADA-GEL and HPL hydrogels were chosen in contrast to Pluronic F-127 to be ideal hydrogels, serving as a printing substrates. In this context, it should also be mentioned that ADA-GEL is a very flexibly modifiable bioink for various biomedical applications [48,49] and that it can even be synthesised in an electrically conductive way using polypyrole [50]. In future studies, the chemical flexibility of PEG moieties in ADA-PEG(+) bioinks will be exploited allowing the covalent introduction of ECM components into the hydrogel, which showed proper extra cellular biomimicry within the bioink droplet regardless of the printing substrate [36,51]. The use of alginate based hydrogels modified with adhesive linkers for DoD allows, thus, the generation of a novel hybrid bioink leading to highly controlled and reproducible cell response depending on the chosen linkers, thus enabling novel applications of targeted cell DoD printing. The targeted printing of these developed bioinks on modifiable substrates, e.g., electrically conductive chips, will also be an important part of future work. This specifically facilitates the electrical stimulation of printed cells on chips providing a smart application of targeted DoD printing of cells at predefined locations.

## 4. Materials and Methods

Chemicals and solvents were purchased from the following suppliers and used if not otherwise noted as received.

Sodium alginate (PH163S2, VIVAPHARM^®^, JRS PHARMA GmbH & Co. KG, Rosenberg, Germany) calcium chloride dehydrate (CaCl_2,_ Sigma Aldrich, Germany), sodium (meta)periodate (NaIO_4,_ Sigma Aldrich, Germany), ethylene glycol, ethanol (Sigma Aldrich, Germany), gelatin (GEL, Type A, porcine skin, Bloom 300, Sigma Aldrich, Germany), human platelet lysate (HPL, PL-BioScience, Germany), Pluronic F-127 (Sigma Aldrich, Germany), Dulbecco’s phosphate buffered saline (DPBS, Sigma Aldrich, Germany), Hanks balanced salt solution (HBSS, Sigma Aldrich, Germany), Dulbecco’s modified eagle medium (DMEM, Sigma Aldrich, Germany), linear polyethylene glycols with two hydroxy end groups (PEG-diol) with different molecular weights of 1000 g·mol^−1^ (PEG-diol 1 kDa, ABCR), 4000 g·mol^−1^ (PEG-diol 4 kDa, Sigma Aldrich, Germany) and 8000 g·mol^−1^ (PEG-diol 8 kDa, Sigma Aldrich, Germany), triethylamine (Et_3_N, Sigma Aldrich, Germany), anhydrous dichloromethane (DCM, Sigma Aldrich, Germany), *p*-toluenesulfonyl chloride (TsCl, ABCR, Germany), ammonia 25 wt.% in water (VWR Chemicals, Germany), isopropanol (VWR Chemicals, Germany), diethyl ether (Fisher Chemical, Germany), ammonium chloride (Grüssing, Germany), sodium sulphate (Roth, Germany), hydrochloric acid (HCl, Merck, Germany), tetrahydrofuran (THF, Acros Organics, Germany), 2,4,6-trinitrobenzoenesulfonate (TNBS, Sigma Aldrich, Germany), sodium bicarbonate (Sigma Aldrich, Germany), Calcein AM and DAPI (Thermo Fisher, Germany). Fibroblast cell culture medium (DMEM, Thermo Fisher, Germany) was supplemented with 1% pen/strep and 1% glutamine (Thermo Fisher, Germany) and 10% fetal calf serum (BCS, Corning, Germany).

### 4.1. Bioink Synthesis

#### 4.1.1. Synthesis of ADA

ADA was synthesized by the oxidation of alginate using sodium (meta)periodate (NaIO_4_) in a solvent consisting of water and ethanol (50:50) according to the protocol of Genc et al., with slight modifications [26]. Briefly, 1.34 g NaIO_4_ were dissolved in deionized (DI, Direct-Q^®^, Merck Millipore, Germany) water and added under dark conditions to an alginate dispersion consisting of 10.0 g alginate in ethanol. This reaction mixture was vigorously stirred for 6 h under light absence and subsequently quenched by the addition of 10.0 mL of ethylene glycol under continuously stirring. After 30 min, the reaction mixture was left to rest for 10 min, enabling the formation of two phases, whereas the upper aqueous phase was slowly decanted. Then, the resultant ADA suspension was dissolved in 400 mL DI water and subsequently dialyzed (MWCO: 6–8 kDa, Spectrum LAB, USA) in darkness for further 4 days with daily water exchanges and finally lyophilised.

#### 4.1.2. Synthesis of PEG-Diamine

The synthesis of the linear polyethylene glycols with two amino end groups (PEG-diamine) was performed in analogy to the two-step procedure reported in the literature [52,53,54,55]. Exemplary, the synthesis of PEG-diamine with a molecular weight of 4 kDa is explained in the following.

Prior to the reaction PEG-diol was dried overnight under high vacuum at 60 °C. In the first step, the hydroxy end groups of the PEG-diol were activated with *p*-toluenesulfonyl chloride (TsCl) to prepare the PEG-ditosylate intermediates. Thereafter, PEG-diol (250 g, 0.0625 mol) and *p*-toluenesulfonyl chloride (TsCl) (35.75 g, 0.1875 mol) were dissolved in 400 mL anhydrous dichloromethane (DCM) followed by dropwise addition of triethylamine (Et_3_N, 26.0 mL, 18.97 g, 0.1875 mol) at 0 °C under argon atmosphere. The resulting mixture was stirred at room temperature overnight. The organic solution was washed six times with water. In case of a poor phase separation, ammonium chloride can be added. After separation of the aqueous and organic phase, the organic phase was dried over sodium sulphate and concentrated under reduced pressure. After precipitation in 1.50 L cold diethyl ether (0 °C), the PEG-ditosylate was obtained as a white powder and dried under high vacuum overnight. An additional purification step was carried out by dissolving the intermediate in DCM and extracting three additional times with water. The DCM phase was dried over sodium sulphate, concentrated, and precipitated in cold diethyl ether (0 °C). The overall yield was about 178 g (65%). In the second step, the tosylate end groups were converted with ammonia to amino end groups. For this, PEG-ditosylate (177.6 g, 0.0412 mol) was dissolved in 700 mL 25 wt.% aqueous ammonia solution. The reaction mixture was stirred for 48 h at room temperature. The reaction mixture was dissolved in DCM and washed five times with water. The DCM phase was dried over sodium sulphate and concentrated under reduced pressure. PEG-diamine 4 kDa was isolated by precipitation in 2 L cold diethyl ether (0 °C) and dried under high vacuum over night, yielding a white powder. Yield of 154 g (93%) were obtained. The two other PEG-diamines (molecular weight = 1 kDa and 8 kDA) were synthesized in the same manner.

#### 4.1.3. Preparation of ADA-PEG

To facilitate homogenous hydrogel solutions, dried ADA with a degree of oxidation (%DO) of 13% was dissolved in Dulbecco’s phosphate buffered saline (DPBS) and stirred overnight, yielding a 5% (*w*/*v*) solution. Further, solid PEG-diol (molecular weight = 1–8 kDa) as well as PEG-diamine (molecular weight = 1–8 kDa) were dissolved in DPBS yielding PEG solutions. The concentration of each solution was determined so that all aldehyde groups present in ADA were sufficiently intercepted by possible amine groups present in PEG-diamines. Values for PEG-diols were derived from the corresponding PEG-diamine ones. In Table S1, detailed information about the used concentrations can be found. Then, all PEG solutions were combined with ADA solutions in a ratio of 1:1 yielding 4 mL inks and stirred for 10 min at 37 °C, respectively, yielding either a hydrogel system with (+) or without (-) Schiff’s base formation, namely, ADA-PEG(+) or ADA-PEG(-) (Table 2). For cell experiments, all hydrogels were additionally sterile filtered using Millipore filters (Rotilabo-syringe filters, PDVDF, Carl Roth, Germany) with pore diameters of either 0.22 µm (for PEG solutions) or 0.45 µm (for ADA solutions).

### 4.2. Bioink Characterization

#### 4.2.1. Chemical Composition

^1^H-NMR spectra were recorded using a Avance 300 spectrometer (Bruker, Germany) with an operating frequency of 300 MHz using deuterated chloroform (CDCl_3_) as solvent to confirm the successful conversion from PEG-diol to PEG-diamine. A standard procedure of potentiometric titration was performed to determine M_n_ of the synthesized PEG-diamines using a Solvotrode easyClean electrode (Metrohm, Germany). For this, the respective PEG-diamine (around 0.50 g) was dissolved in 25.0 mL THF and then 25.0 mL isopropanol was added. The titration of the amino end groups was conducted by adding a standard titrant of 0.1 M hydrochloric acid (HCl) in isopropanol. Every titration was conducted three times (*n* = 3) and *M*_n_ was determined by using following equation:

(1) $${\bar{M}}_{n}=\frac{m_{s}\cdot z}{V_{eq}\cdot c}$$

where *m_s_* is the weighted mass of PEG-diamine and *z* the number of amine end groups. *V_eq_* describes the volume of added 0.1 M HCl solution until the equivalent point is reached and *c* is the concentration of hydrochloride acid solution.

Fourier transform infrared spectroscopy with an attenuated total reflectance technology (ATR-FTIR) was performed for the chemical characterization of ADA-PEG inks using an IRAffinity-1S Fourier Transform Infrared Spectrometer (Shimadzu, Japan). All samples were measured as dry products after lyophilisation. Furthermore, all spectra were recorded in the mode of absorbance using 40 scans and a resolution of 4 cm^−1^.

Moreover, to investigate the chemical structure of all ADA-PEG(+), ^13^C-solid state nuclear magnetic resonance (^13^C-solid state NMR) spectroscopy was performed using ADA, PEG-diamin 4 kDa and ADA-PEG(+) 4 kDa. All products were firstly lyophilized and subsequently packed into zirconia rotors (100 mg/sample). Afterwards, these rotors were spun at 10 kHz using a Bruker Avance spectrometer (Bruker Biospin GmbH, Germany), whereas a Larmor frequency of 100 MHz on the nuclei of ^13^C atoms were operated, respectively [56].

#### 4.2.2. Degree of Crosslinking

For the determination of adjustment of the equilibrium of the degrees of crosslinking between ADA and PEG-diamine moieties, the trinitrobenzoic acid (TNBS) assay according to Nguyen et al. was slightly changed and performed [57]. In brief, ADA-PEG(+) hydrogels with different PEG chain lengths (1–8 kDa) were reacted (1:1) for 10, 30 or 60 min at 37 °C and subsequently frozen at −20 °C followed by lyophilization. Afterwards, 5 replicates containing 5.00 mg hydrogel were weighted and dissolved in 1 mL of a sodium bicarbonate buffer solution (4%) at 60 °C, combined with 1 mL of a TNBS solution (0.5%) and incubated at 60 °C for 4 h, leading to an orange colour change in the solution. After mixing 1 mL of the reaction mixture with 3 mL of HCl (6 M), the solution was incubated for further 1.5 h at 40 °C. Finally, the yellow solutions were diluted 10 times and the absorption at 346 nm was read against the blank solution using an UV/Vis spectrometer, respectively. PEG-diamine was chosen as a reference and treated like the sample solutions, with the difference that HCl was added prior the addition of TNBS.

The decrease in absorbance from PEG-diamine to ADA-PEG samples can be linked to the degree of crosslinking, calculated by the following equation:

(2) $$\%Crosslinking=\frac{A_{PEG-diamine}-A_{ADA-PEG}}{A_{PEG-diamine}}*100$$

With *A_PEG-diamine_* as the absorbance of *PEG-diamine* used as reference and *A_ADA-PEG_* as the absorbance of the sample. As a negative control group ADA-PEG(-) 1 kDa was tested.

#### 4.2.3. Rheological Characterization

The rheological measurements were conducted directly after the preparation of chemically crosslinked (+) and uncrosslinked (-) ADA-PEG hydrogels with different PEG chain lengths (1–8 kDa) at 37 °C using a shear rheometer (AR G-2, TA instruments, USA). Temperature control was ensured using a Peltier-element. Sample-drying was minimized by the equipped solvent trap, filled with DPBS. The used plate–plate geometry was 40 mm in diameter and a measurement gap of 500 µm was used for all experiments. After samples were transferred to the rheology plate, the platform was firstly moved to 525 µm and excessive material was removed before decreasing the gap to 500 µm. To determine possible crosslinking processes, the storage modulus (G′) and the loss modulus (G″) were characterized through oscillatory time sweep experiments. The shear stress and frequency were kept constant at 0.1 Pa and 1 rad·s^−1^, respectively. To access the zero-shear viscosity of tested samples, a flow sweep ranging from 1 s^−1^ to 100 s^−1^ was conducted.

#### 4.2.4. In Vitro Degradation and Swelling Behaviour

Weight gain and loss studies were performed to investigate the swelling and degradation behaviour of chemically crosslinked (+) and uncrosslinked (-) ADA-PEG hydrogels with different PEG chain lengths (1–8 kDa). Hydrogel films were produced under sterile conditions using a sterilized round shaped puncher (Ø = 10 mm) after the hydrogels were crosslinked with calcium chloride (CaCl_2_) for 10 min, as described by Sarker et al. [12]. All films were placed in cell inserts (Invitrogen, Germany) and weighted including cell inserts under sterile conditions prior to immersing them in medium, recording the initial weight and subsequently incubated in Gibco Dulbecco’s Modified Eagle Medium (DMEM) at 37 °C (5% CO_2_, 95% relative humidity) over a period of 21 days. Every composition contains 6 replicates, respectively, and was stored in 6 well plates, including the cell inserts, with medium changes every 3 days. After certain time points, the cell inserts including hydrogel films were dried with sterile tissue paper and weighted using a balance revealing either a swelling (>100%) or degradation (<100%) of materials calculated as follows:

(3) $$W\%=\frac{W_{t}-W_{0}}{W_{0}}\;*100$$

In which *W*_0_ is the initial weight, and *W_t_* corresponds to the mass at the time of weighting the film prior to immersion in fresh culture medium. Then, the hydrogel films were retransferred to the well plates and supplemented with 4 mL of fresh culture medium, followed by incubation for subsequent analysis.

#### 4.2.5. Mechanical Analysis Using Compression Tests

Compression tests were performed using an Instron Universal Testing Machine (Instron 3300 Floor Model, Instron^®^ GmbH, Germany) with a 100 N force measuring cell, to determine a stiffness of chemically crosslinked (+) and uncrosslinked (-) ADA-PEG hydrogels with different PEG chain lengths (1–8 kDa). Hydrogel films were prepared under sterile conditions, as described previously, using a sterilized round shape puncher (Ø = 1.2 mm) and incubated over 21 days at 37 °C (5% CO_2_, 95% relative humidity) with medium changes every 3 days. The loading rate of the cross tool was 0.25 mm·min^−1^ without preloading, whereas the starting distance was set to 1.5 mm at the first point of contact with the material. All trials contained 4 replicates per composition. The modulus of elasticity was determined from the slope of the force/deformation curve at 10% of stress.

### 4.3. Preparation of Hydrogels Used as Printing Substrates

ADA-GEL was prepared according to Genc et al. [26] by mixing a 5% (*w*/*v*) ADA solution with 5% (*w*/*v*) GEL in a ratio of 1:1 for 10 min at 37 °C to facilitate a sufficient crosslinking between ADA and GEL yielding ADA-GEL. Wells used for printing were then filled with 2 mL of the hydrogel and stored for 30 min at 5 °C to thermally gel ADA-GEL. Furthermore, HPL was prepared by mixing 9 mL of pure DMEM with 1 mL PL-Matrix solution and subsequently transferred into the printing well so that every well contained 2 mL of HPL. After 24 h of incubation at 37 °C (5% CO_2_, 95% relative humidity), the gelation of HPL was accomplished. Lastly, to obtain Pluronic F-127 hydrogels, solid Pluronic F-127 powder was dissolved in ultrapure water by storing the dispersion at 5 °C overnight to obtain a 20% (*w*/*v*) solution. Then, 2 mL of the hydrogel was transferred into the printing wells.

### 4.4. Cytocompatibility

All chemically crosslinked (+) and uncrosslinked (-) ADA-PEG hydrogels were prepared, as described previously. Then, cell suspensions containing NIH/3T3 cells (1 Mio·mL^−1^) were centrifuged for 5 min at 350 rpm. After the supernatant solutions were removed, previously prepared hydrogels were transferred to the cell pallets and mixed smoothly using a high viscous pipette (MICROMAN E, Gilson, USA), respectively. Subsequently, all bioinks were transferred to well plates and all scaffolds were treated with CaCl_2_ for 10 min, enabling a strong crosslinking of the polymer network followed by the immersion in DMEM supplemented with 1% pen/strep and 1% glutamine and 10% fetal calf serum. After 24 h the medium was replaced by HBSS and stained using master stock solutions containing Calcein (4 µL·mL^−1^) or DAPI (1 µL·mL^−1^) diluted in HBSS, respectively, where the procedure of the manufacturer (ThermoFisher, Germany) was fully adapted. The epifluorescence images were recorded using a fluorescence microscope (Axio, Zeiss, Germany). Finally, the software “ImageJ” (ImageJ software version 1.52n) and the plugin “ITCN” were used to count the number of living and total cells.

### 4.5. Evaluation of the Printing Process

For the DoD approach, microvalve cell printing was realized using a BioX 3D printer (Cellink, Sweden). This technique allows to eject droplets using an electromagnetic droplet (EMD) print head tool and a microvalve (diameter: 100 µm or 300 µm). The printer was housed in a biosafe cabinet and sterilised using UV light.

#### 4.5.1. Calculation of Shear Stress during Printing

Based on studies of Blaeser et al. [44], shear stresses in the needle during the printing process of the BioX printer equipped with an EMD print head was empirically determined for each bioink. First of all, based on shear rates up to 1000 s^−1^, the consistency factor *k* and the flow exponent *n*, that describe the flow behaviour of non-Newtonian fluids according to the Ostwald-de Waele relation, were determined for the ADA-PEG(+) formulations. Next, the drop ejection speed as well as the average nozzle shear stress were calculated according to the previously presented algorithm [44]. Besides the consistency factor and flow exponent, the nozzle diameter (300 µm), the static pressure (50 kPa), the height of the fluid column within the liquid reservoir (8.5 cm) as well as the material density (1.3 g·mL^−1^) were applied for the analytical estimation.

#### 4.5.2. Evaluation of Printing Parameters

To analyse the working principle and functioning of the printer, the impact of set parameters on a predefined geometry of the printed scaffold was investigated. For this purpose, 2 mL of each ADA-PEG(+) hydrogel was transferred into a cartridge placed into the EMD tool which was fitted with a microvalve (Ø = 100 or 300 µm). In a first attempt, the influence of printing parameters on the distances of ejected droplets was evaluated and the 300 µm microvalve was used to print one layer of a square rectangle with dimensions of 10 mm × 10 mm. While printing parameters, e.g., pressure range, printing speed, printing height and open time, were kept constant for all compositions, the cycle time was varied (Table S2). As ink covalently crosslinked ADA-PEG(+) hydrogels with different chain lengths (1–8 kDa) were used to print the described structure under the variation of cycle time (50–1000 ms). After printing six replicates of the desired scaffold, the ImageJ software and Fiji plugin were used for the visual analysis of the camera images. Using these images, distances between droplets as well as deviation of printed areas from the simulated ones were determined for each ink, which gave a deep insight into the working principle as well as the printing accuracy of the used DoD printer.

To evaluate the printing precision, the filament fusion test according to Cai et al. [45], with slight modifications in the data evaluation, was performed. Firstly, one layer of the meandering pattern was printed using ADA-PEG(+) inks, a pressure range of 30–40 kPa, a printing speed of 5 mm·s^−1^, a cycle time of 400 ms, an open time of 1 ms and a constant printing height of 5 mm for all ADA-PEG(+) (1–8 kDa) inks, respectively (*n* = 2). Light microscopy images of printed scaffolds were taken using the Axio Scope A1 light microscope (PrimoVert, Carl Zeiss, Germany) and evaluated using the ImageJ software. Depending on the distances between the parallel struts, the meandering pattern was classified into five different segments, with segment 1 exhibiting 2000 µm as strand distance, segment 2 having 1500 µm, segment 3 showing 1000 µm, segment 4 exhibiting 750 µm and segment 5 having 500 µm as a strand distance. Printing accuracy of the 100 µm and 300 µm microvalve was determined by measuring the corresponding strand distances of all segments for all ADA-PEG(+) inks.

In a last approach, the cell distribution within single droplets and the determination of final cell concentrations per droplet were investigated. For this purpose, NIH/3T3 cells in different concentrations (100,000–10 Mio·mL^−1^) were combined with ADA-PEG(+) 1 kDa, respectively, and printed on PS. This was repeated for the 100 µm and for the 300 µm microvalve integrated into the EMD printing head. Subsequently, light microscopy images of the individual droplets were taken, which were then analysed by ImageJ in terms of droplet diameter and cell number per droplet (*n* = 20).

#### 4.5.3. Cell Printing

To choose the ideal bioink as well as the most suitable printing substrate, NIH/3T3 cells were embedded in ADA-PEG(+) hydrogels and subsequently transferred into cartridges, respectively. Then, one layer of a square rectangle with dimensions of 10 mm × 10 mm was printed on a layer consisting of PS, Pluronic F-127, ADA-GEL or HPL. After printing, all scaffolds were crosslinked with CaCl_2_ (10 min) and then incubated in cell culture medium at 37 °C for 24 h. Then, all cells within the bioinks on the printing substrates were dyed using Calcein AM and DAPI according to Genc et al. [26]. Cell viability was calculated using fluorescence images (*n* = 9) and ImageJ. Lastly, cell distribution in individual droplets were investigated using a microscope setup consisted of a Nikon Eclipse Ni-E which was equipped with an A1R HD Scan head for Confocal Imaging (Nikon NiE A1RHD confocal, Nikon, Japan).

### 4.6. Statistical Analysis

One way analysis of variance (ANOVA) was conducted for the analysis of the mean differences using the Origin 2019 software (OriginLab Coporation, USA). A certain number of replicates (*n*) was examined for each experiment. The result was shown as means of ± standard deviation (SD) and the level of statistical significance was determined with * *p* < 0.05, ** *p* < 0.01 and *** *p* < 0.001 intervals of confidence.

## Figures and Tables

**Table 1:** Rheological characterization of different ADA-PEG hydrogel formulations with varying molecular weights and analytical modulation of their printing behaviour.

	ADA-PEG(+) 1 kDa	ADA-PEG(+) 4 kDa	ADA-PEG(+) 8 kDa
Consistency factor *k* (mPa·s)	20.90	26.79	43.41
Flow exponent *n*	0.96	0.98	0.97
Drop ejection speed (m·s^−1^)	7.35	6.61	5.94
Average nozzle shear stress (kPa)	1.3	1.9	2.3

**Table 2:** List of used ADA-PEG inks and information about their components consisting of ADA with a degree of oxidation of 13% and different PEG types (molecular weight = 1–8 kDa).

Label of Ink	ADA Type	PEG Type
ADA-PEG(+) 1 kDa	%DO = 13%	PEG-diamine 1 kDa
ADA-PEG(+) 4 kDa	%DO = 13%	PEG-diamine 4 kDa
ADA-PEG(+) 8 kDa	%DO = 13%	PEG-diamine 8 kDa
ADA-PEG(-) 1 kDa	%DO = 13%	PEG-diol 1 kDa
ADA-PEG(-) 4 kDa	%DO = 13%	PEG-diol 4 kDa
ADA-PEG(-) 8 kDa	%DO = 13%	PEG-diol 8 kDa

## Data Availability

Data supporting reported results will be provided upon request.

## Supplementary Materials

The following supporting information can be downloaded at: https://www.mdpi.com/article/10.3390/gels8040206/s1, Figure S1: Two-step synthesis of PEG-diamine. First, PEG-diol was reacted to tosylate end groups with p toluenesulfonyl chloride (TsCl) and converted in a second step to amino end groups to PEG-diamines using ammonia solution at RT; Figure S2: Comparison of the 1H-NMR spectra of PEG-ditosylate 4 kDa and PEG-diamine 4 kDa; Figure S3: Exemplary light microscopy images of ejected droplets consisting of ADA-PEG(+) 1 kDa loaded with NIH/3T3 cells (1–10 mio·mL^−1^) and printed with two different nozzle diameters: 100 µm (top) and 300 µm (bottom); Figure S4: Diagram showing the average diameter of printed ADA-PEG(+) 1 kDa droplets using the 100 µm and the 300 µm nozzle, respectively Average was calculated from 6 individual printing experi-ments with different initial cell concentrations (100.000–10 mio·mL^−1^). ns *p* < 0.05, * *p* < 0.01, *** *p* < 0.001; Table S1: List of covalently crosslinked (+) and uncrosslinked (-) ADA-PEG bioinks and information about their ADA and PEG-diamine or PEG-diol concentrations for the preparation of final bioinks after the combination of ADA and PEG hydrogels in a ratio of 1:1; Table S2: List of parameters used for the DoD printing of ADA-PEG(+) bioinks.
