# Supplementary material for: Targeted Printing of Cells: Evaluation of ADA-PEG Bioinks for Drop on Demand Approaches
Source: Gels. 2022 Mar 24;8(4):206. doi: 10.3390/gels8040206 (PMC9032277; doi:10.3390/gels8040206)
Supplement: Supplementary file 1 [file gels-08-00206-s001.zip › gels-1599642-supplementary.pdf]

gels

Article

Targeted Printing of Cells: Evaluation of ADA-PEG Bioinks for Drop on Demand Approaches

Emine Karakaya [1], Faina Bider [1], Andreas Frank [2], Jörg Teßmar [3], Lisa Schöbel [1], Leonard Forster [3], Stefan Schrüfer [4], Hans-Werner Schmidt [2], Dirk Wolfram Schubert [4,5], Andreas Blaeser [6,7], Aldo R. Boccaccini [1] and Rainer Detsch [1,*]

* Correspondence: rainer.detsch@fau.de; Tel.: +49-9131-85-69611

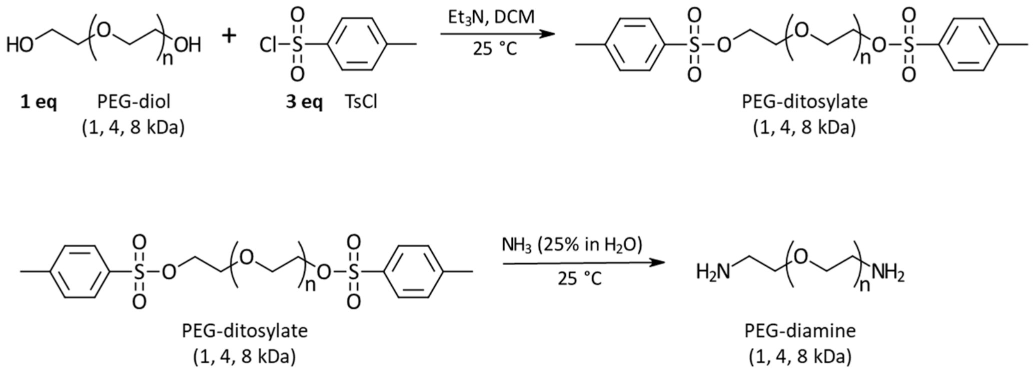

Figure S1. Two-step synthesis of PEG-diamine. First, PEG-diol was reacted to tosylate end groups with *p*-toluenesulfonyl chloride (TsCl) and converted in a second step to amino end groups to PEG-diamines using ammonia solution at RT.

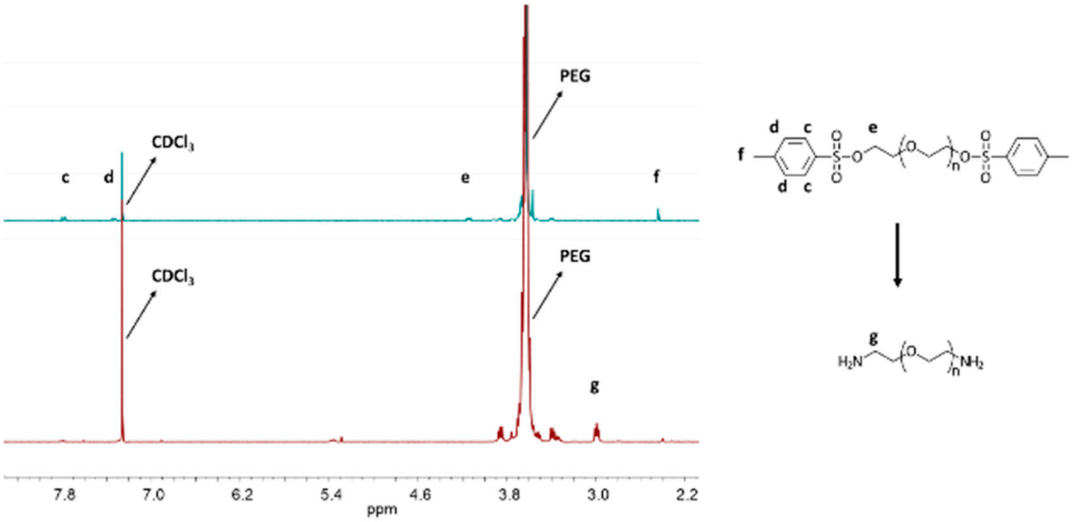

Figure S2. Comparison of the ^{1}H-NMR spectra of PEG-ditosylate 4 kDa and PEG-diamine 4 kDa.

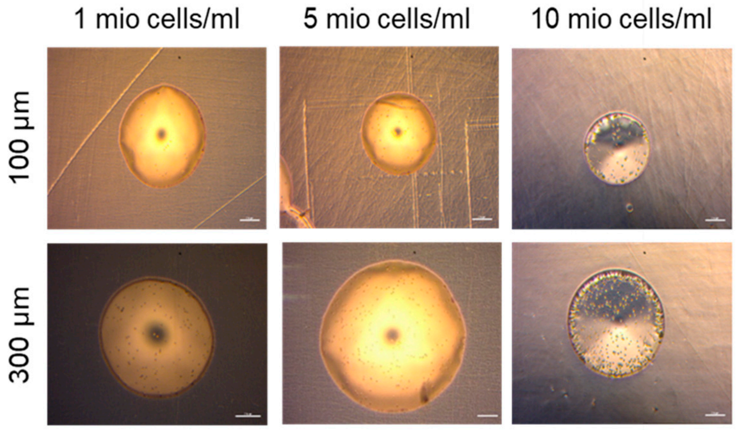

Figure S3. Exemplary light microscopy images of ejected droplets consisting of ADA-PEG(+) 1 kDa loaded with NIH/3T3 cells (1 - 10 mio·mL^{-1}) and printed with two different nozzle diameters: 100 µm (top) and 300 µm (bottom).

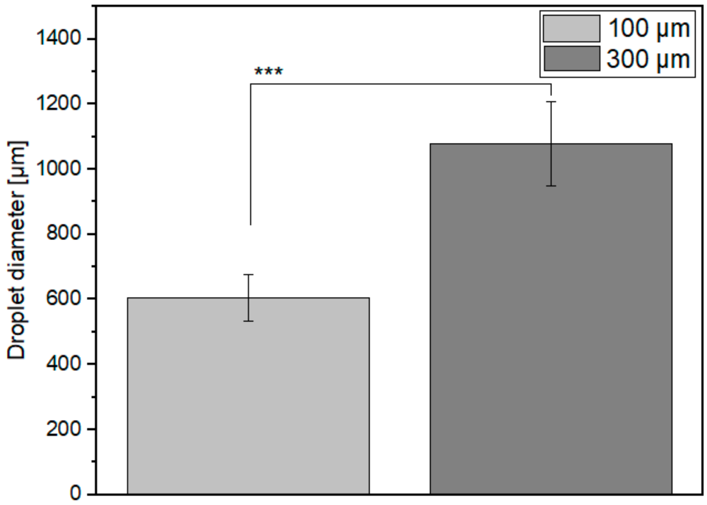

Figure S4. Diagram showing the average diameter of printed ADA-PEG(+) 1 kDa droplets using the 100 µm and the 300 µm nozzle, respectively Average was calculated from 6 individual printing experiments with different initial cell concentrations (100.000 – 10 mio·mL^{-1}). ns *$p < 0.05$, **$p < 0.01$, ***$p < 0.001$.

Table S1. List of covalently crosslinked (+) and uncrosslinked (-) ADA-PEG bioinks and information about their ADA and PEG-diamine or PEG-diol concentrations for the preparation of final bioinks after the combination of ADA and PEG hydrogels in a ratio of 1:1.

Bioink label*	Concentration of ADA for 2 mL (w/v%)	Concentration of PEG-diamine for 2 mL (w/v%)	Concentration of PEG-diol for 2 mL (w/v%)
ADA-PEG(-) 1 kDa	5.00	-	1.00
ADA-PEG(-) 4 kDa	5.00	-	4.25
ADA-PEG(-) 8 kDa	5.00	-	8.65
ADA-PEG(+) 1 kDa	5.00	1.00	-
ADA-PEG(+) 4 kDa	5.00	4.25	-
ADA-PEG(+) 8 kDa	5.00	8.65	-

* Final volume: 4 mL.

Table S2. List of parameters used for the DoD printing of ADA-PEG(+) bioinks.

Bioink label	Printing pressure [kPa]	Printing speed [mm·s^{-1}]	Printing height [mm]	Open time [ms]	Cycle time [ms]
ADA-PEG(+) 1 kDa	15 - 40.0	5.00	10.0	1.00	50 - 1000
ADA-PEG(+) 4 kDa	15 - 40.0	5.00	10.0	1.00	50 - 1000
ADA-PEG(+) 8 kDa	15 - 40.0	5.00	10.0	1.00	50 - 1000
